# Supplementary material for: A pseudovirus system enables deep mutational scanning of the full SARS-CoV-2 spike
Source: bioRxiv. 2022 Oct 13:2022.10.13.512056. Preprint. [Version 1] doi: 10.1101/2022.10.13.512056 (PMC9580381; doi:10.1101/2022.10.13.512056)
Supplement: 1 [file NIHPP2022.10.13.512056V1-supplement-1.pdf]

bioRxiv preprint doi: https://doi.org/10.1101/2022.10.13.512056; this version posted October 13, 2022. The copyright holder for this preprint (which was not certified by peer review) is the author/funder, who has granted bioRxiv a license to display the preprint in perpetuity. It is made available under aCC-BY 4.0 International license.

Supplementary Figure 1

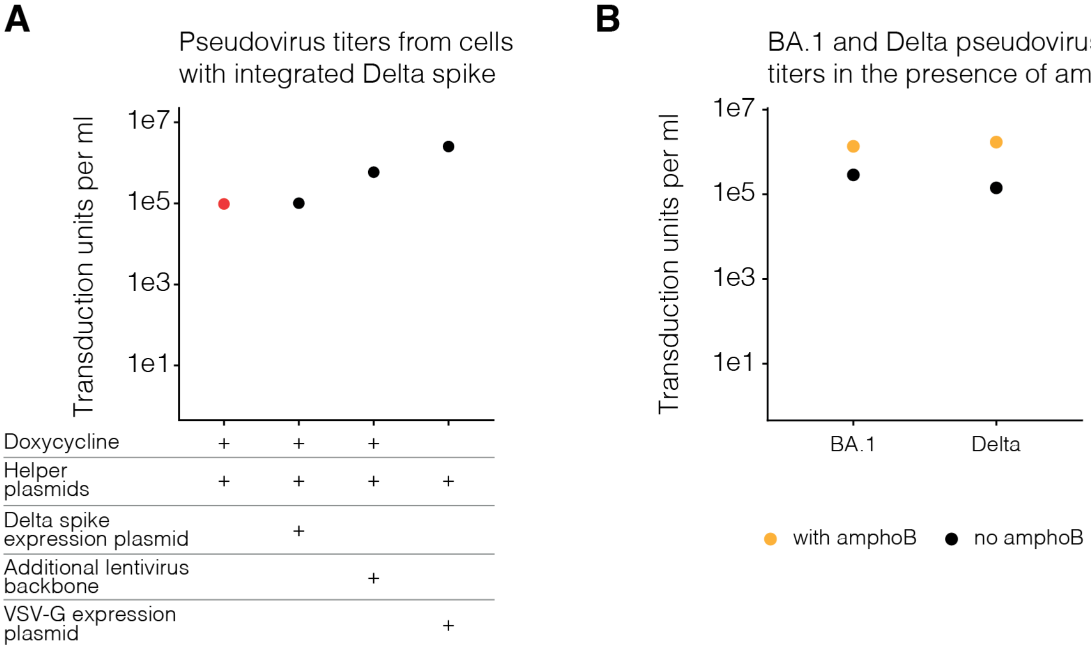

Supplementary Figure 1. Pseudovirus titers from phenotype-genotype linked lentiviruses. **(A)** Delta spike pseudotyped lentivirus titers. Viruses were produced under indicated conditions from cells with integrated lentivirus genomes carrying Delta spike. Virus titers for conditions used to generate the actual deep mutational scanning libraries are coloured red. Viruses were titrated on ACE2-TMPRSS2-HEK-293T cells. **(B)** BA.1 or Delta spike-pseudotyped lentivirus titers in the presence or absence of amphotericin B (amphoB). BA.1 virus was titrated on ACE2-HEK-293T cells and Delta virus was titrated on ACE2-TMPRSS2-HEK-293T cells.

bioRxiv preprint doi: https://doi.org/10.1101/2022.10.13.512056; this version posted October 13, 2022. The copyright holder for this preprint (which was not certified by peer review) is the author/funder, who has granted bioRxiv a license to display the preprint in perpetuity. It is made available under aCC-BY 4.0 International license.

Supplementary Figure 2

A

variant library	*number of barcoded variants*
Delta Lib-1	49,053
Delta Lib-2	48,855

B

variant library	*intended number of mutations*	*mutations in the library(% intended)*
Delta	6,852	6,771(98.8)

C

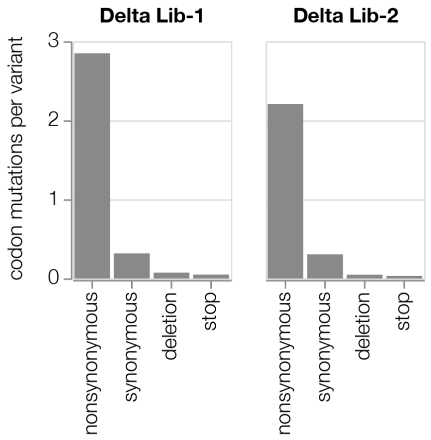

D

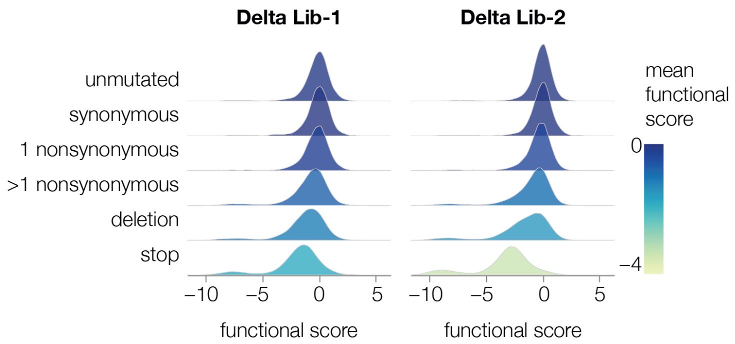

Supplementary Figure 2. Delta spike deep mutational scanning libraries

(A) Total number of barcoded variants in each Delta library. **(B)** Coverage of intended mutations across both Delta libraries. **(C)** Average number of mutations per barcoded spike in Delta libraries. **(D)** Distribution of functional scores for variants with different types of mutations in the Delta libraries.

bioRxiv preprint doi: https://doi.org/10.1101/2022.10.13.512056; this version posted October 13, 2022. The copyright holder for this preprint (which was not certified by peer review) is the author/funder, who has granted bioRxiv a license to display the preprint in perpetuity. It is made available under aCC-BY 4.0 International license.

Supplementary Figure 3

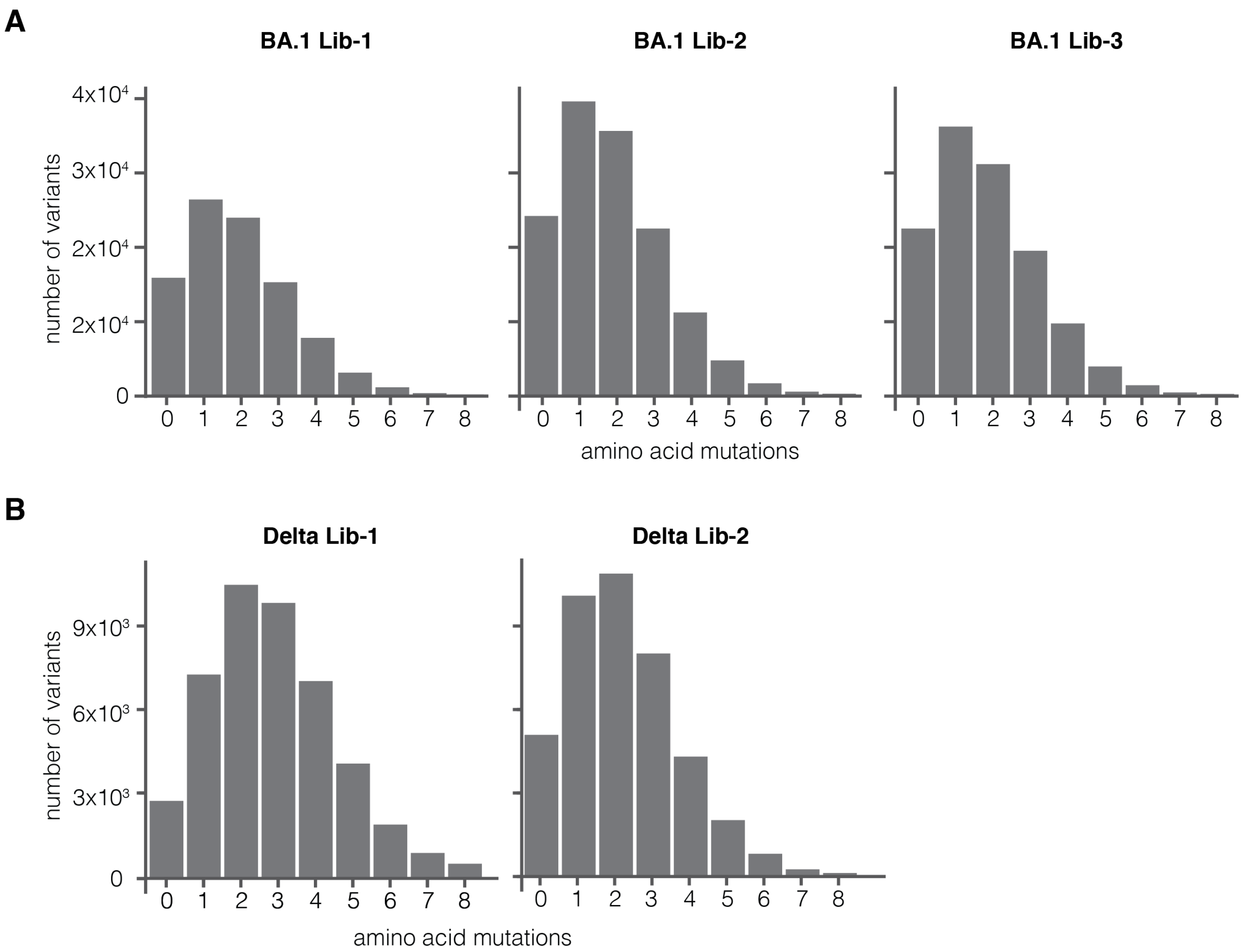

Supplementary Figure 3. Distribution of the number of amino-acid mutations per variant in BA.1 (A) and Delta (B) deep mutational scanning libraries

bioRxiv preprint doi: https://doi.org/10.1101/2022.10.13.512056; this version posted October 13, 2022. The copyright holder for this preprint (which was not certified by peer review) is the author/funder, who has granted bioRxiv a license to display the preprint in perpetuity. It is made available under aCC-BY 4.0 International license.

Supplementary Figure 4

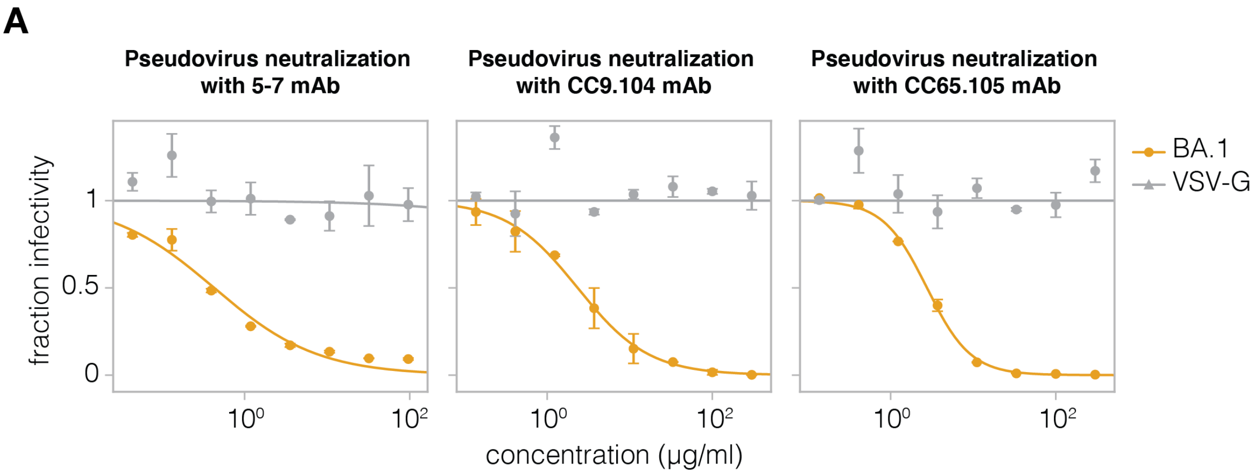

Supplementary Figure 4. The VSV-G neutralization standard is not neutralized by antibodies 5-7, CC9.104 and CC65.105.

(A) Neutralization assays using NTD-targeting 5-7 mAb and S2-targeting CC9.104 and CC65.105 antibodies against lentivirus pseudotyped with BA.1 spike or VSV-G.

bioRxiv preprint doi: https://doi.org/10.1101/2022.10.13.512056; this version posted October 13, 2022. The copyright holder for this preprint (which was not certified by peer review) is the author/funder, who has granted bioRxiv a license to display the preprint in perpetuity. It is made available under aCC-BY 4.0 International license.

Supplementary Figure 5

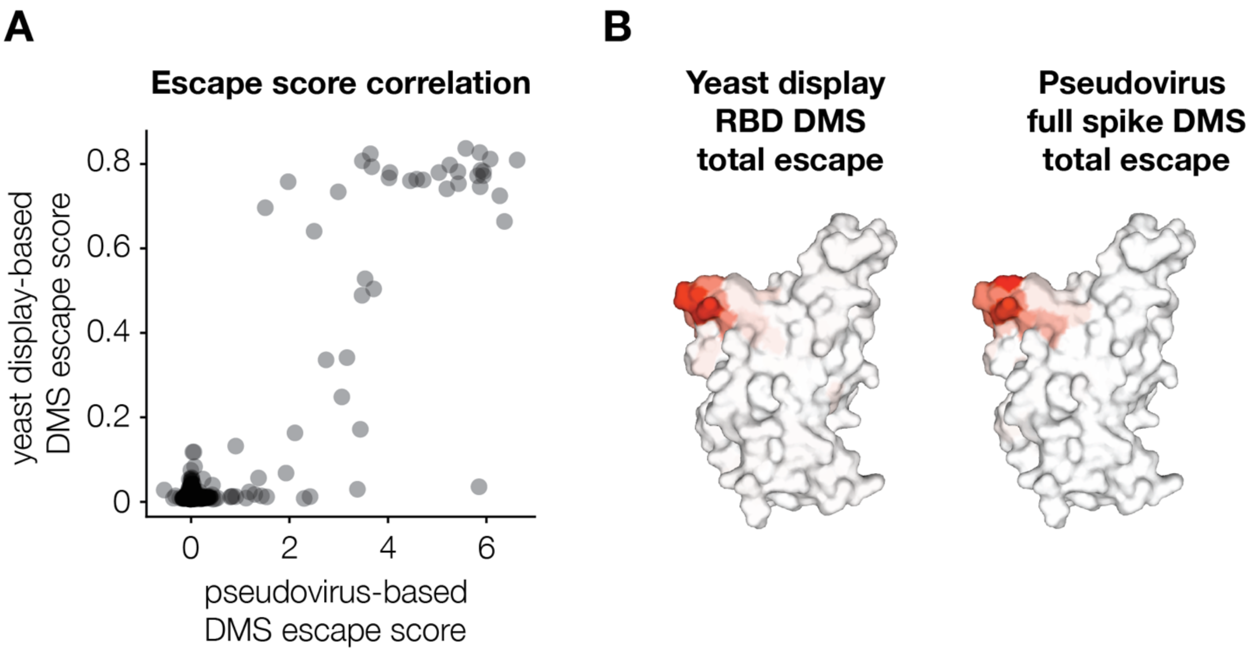

Supplementary Figure 5. Comparison between LY-CoV1404 escape mapping using full spike pseudovirus deep mutational scanning versus our previously described yeast-display deep mutational scanning of just the RBD.

(A) Correlation between measured mutation-level escape scores for LY-CoV1404 antibody in pseudovirus and yeast display deep mutational scanning experiments. Yeast display data is taken from (Starr et al., 2022). **(B)** Surface representation of SARS-CoV-2 RBD coloured by sum of escape scores at that site. PDB ID: 6XM4.

bioRxiv preprint doi: https://doi.org/10.1101/2022.10.13.512056; this version posted October 13, 2022. The copyright holder for this preprint (which was not certified by peer review) is the author/funder, who has granted bioRxiv a license to display the preprint in perpetuity. It is made available under aCC-BY 4.0 International license.

Supplementary Figure 6

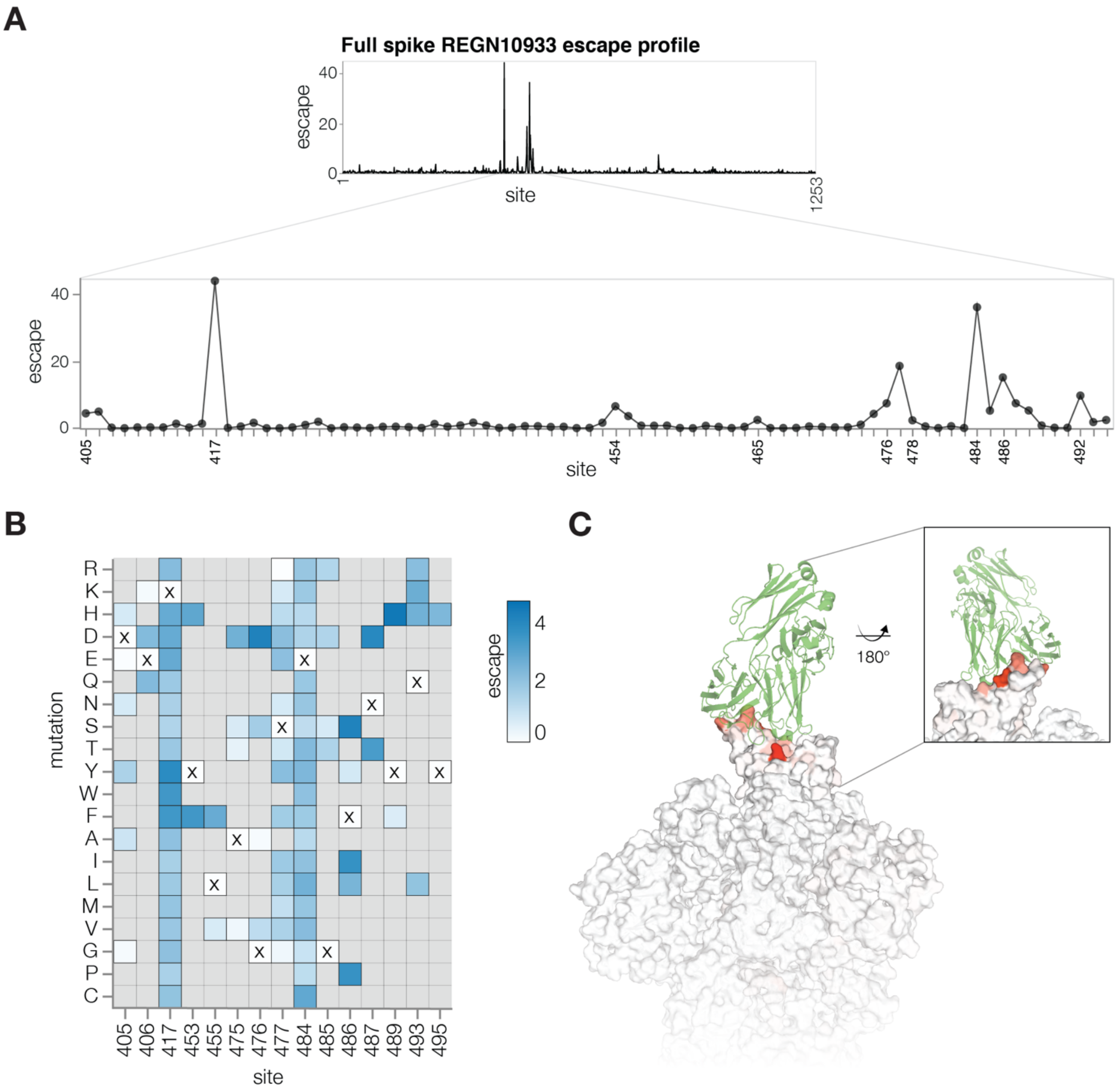

Supplementary Figure 6. Antibody REGN10933 escape mapping using Delta deep mutational scanning libraries

(A) Total escape scores for each site within Delta spike and a zoomed-in plot showing key escape sites. **(B)** Heatmap of mutation escape scores at key sites. Residues marked with X are the wild-type amino acids in Delta sequence. Amino acids not present in our libraries are shown in gray. An interactive version of this heatmap for the entirety of spike is at https://dms-vep.github.io/SARS-CoV-2_Delta_spike_DMS_REGN10933/REGN10933_escape_plot.html **(C)** Surface representation of spike coloured by sum of escape scores at that site. REGN10933 antibody is shown in green. PDB structures 6XDG and 6XM4 were aligned to make this figure. Site numbering in all plots is based on the Wuhan-Hu-1 sequence.

bioRxiv preprint doi: https://doi.org/10.1101/2022.10.13.512056; this version posted October 13, 2022. The copyright holder for this preprint (which was not certified by peer review) is the author/funder, who has granted bioRxiv a license to display the preprint in perpetuity. It is made available under aCC-BY 4.0 International license.

Supplementary Table 1. Primer sequences used for building deep mutational scanning libraries

Name	Sequence 5′-3′	Function
Mutagenesis primers		
BA.1 oPools	https://github.com/dms-vep/SARS-CoV-2_Omicron_BA.1_spike_DMS_mAbs/tree/main/library_design	Primers to add mutations to BA.1 spike
Delta oPools	https://github.com/dms-vep/SARS-CoV-2_Delta_spike_DMS_REGN10933/tree/main/library_design/results	Primers to add mutations to Delta spike
Spike amplification and joining PCR		
VEP_amp_for	CAGCCGAGCCACATCGCTC	For amplifying spike with flanking sequences from lentivirus backbone
3’rev_lib_LinJoin_KHDC	CGGAAGAGCGTCGTGTAGGGAAAG	
Spike barcoding primers		
ForInd_AddBC_2	gcggaactccactaggaacatttctctctcgaaTCTAGANNNNNNNNNNNNNNNNAGATCGGAAGAGCGTCGTGTAGGGAAAGAG	For adding barcodes to spike gene
5'for_lib_bcing	gcacgcgCAGCCGAGCCACATCGCTCA	
Neutralization standard barcoding primers		
5'for_lib_bcing	gcacgcgCAGCCGAGCCACATCGCTCA	For barcoding neutralization satandard
Barcoding primer pool 1:<br>VSVG_BC1<br>VSVG_BC2<br>VSVG_BC3<br>VSVG_BC4	gcggaactccactaggaacatttctctctcgaaTCTAGAtactttactactgcacAGATCGGAAGAGCGTCGTGTAGGGAAAGAG<br>gcggaactccactaggaacatttctctctcgaaTCTAGAggaccattgcgacgtaAGATCGGAAGAGCGTCGTGTAGGGAAAGAG<br>gcggaactccactaggaacatttctctctcgaaTCTAGAcctagccactagatggAGATCGGAAGAGCGTCGTGTAGGGAAAGAG<br>gcggaactccactaggaacatttctctctcgaaTCTAGAatggagggagtctactAGATCGGAAGAGCGTCGTGTAGGGAAAGAG	
Barcoding primer pool 2<br>VSVG_BC5<br>VSVG_BC6<br>VSVG_BC7<br>VSVG_BC8	gcggaactccactaggaacatttctctctcgaaTCTAGAtagtgtaaacgccacgAGATCGGAAGAGCGTCGTGTAGGGAAAGAG<br>gcggaactccactaggaacatttctctctcgaaTCTAGAccaacgcgtgaatcgcAGATCGGAAGAGCGTCGTGTAGGGAAAGAG<br>gcggaactccactaggaacatttctctctcgaaTCTAGAatcgtatccatgggtaAGATCGGAAGAGCGTCGTGTAGGGAAAGAG<br>gcggaactccactaggaacatttctctctcgaaTCTAGAggtcacgtgtctatatAGATCGGAAGAGCGTCGTGTAGGGAAAGAG	
Spike gene amplification for PacBio long-read sequencing		
PacBio_5pri_C_tag1	ctagccattcagagGCAGCCGAGCCACcTCGCTC	Used for

bioRxiv preprint doi: https://doi.org/10.1101/2022.10.13.512056; this version posted October 13, 2022. The copyright holder for this preprint (which was not certified by peer review) is the author/funder, who has granted bioRxiv a license to display the preprint in perpetuity. It is made available under aCC-BY 4.0 International license.

PacBio_3pri_G_tag1	CGCTCAACCAGTACGAGCCGTAAGTTATGTAACGCGGAACTCCACgAGGAAC	amplifying spike and barcode for PacBio sequencing
PacBio_5pri_G_tag2	ctagccattcagagGCAGCCGAGCCACgTCGCTC	
PacBio_3pri_C_tag2	CGCTCAACCAGTACGAGCCGTAAGTTATGTAACGCGGAACTCCACcAGGAAC	
PacBio_5pri_RND2	CTAGCCATTCAGAGGCAGCCGAG	
PacBio_3pri_RND2	CGCTCAACCAGTACGAGCCGTAAGTTATGTAAC	
Illumina barcode sequencing 1st round PCR primers		
IlluminaRnd1_For	CTCTTTCCCTACACGACGCTCTTCCGATCT	Round 1 primers for barcode sequencing
IlluminaRnd1_Rev3	CTGGAGTTCAGACGTGTGCTCTTCCGATCTgtccctattggcgttactatgggaacatacgtc	
Illumina barcode sequencing library round 2 PCR primers		
Rnd2ForUniversal	AATGATACGGCGACCACCGAGATCTACACTCTTTCCCTACACGACGCTCTTCCGATCT	Universal illumina primer and indexing primer, where xxxxxxx indicates i7 index sequence
Indexing primer	CAAGCAGAAGACGGCATACGAGATxxxxxxxxGTGACTGGAGTTCAGACGTGTGCTCTTCCGATCT	
